# Insulin-Like Peptide 3 (INSL3) Serum Concentration During Human Male Fetal Life

**DOI:** 10.3389/fendo.2019.00596

**Published:** 2019-09-04

**Authors:** Steven M. Harrison, Nicol Corbin Bush, Yi Wang, Zachary R. Mucher, Armando J. Lorenzo, Gwen M. Grimsby, Bruce J. Schlomer, Erika E. Büllesbach, Linda A. Baker

**Affiliations:** ^1^Clinical R&D Sequencing Platform, Broad Institute, MIT and Harvard, Cambridge, MA, United States; ^2^PARC Urology, Frisco, TX, United States; ^3^Endocrinology Division, Department of Internal Medicine, The First Affiliated Hospital of Xi'an Jiaotong University, Xi'an, China; ^4^Department of Urology, Memorial Hermann Health System, Houston, TX, United States; ^5^Department of Pediatric Urology, Hospital for Sick Children, Toronto, ON, Canada; ^6^Phoenix Children's Hospital, Phoenix, AZ, United States; ^7^Division of Pediatric Urology, Department of Urology, University of Texas Southwestern Medical Center, Dallas, TX, United States; ^8^Department of Biochemistry and Molecular Biology, Medical University of South Carolina, Charleston, SC, United States; ^9^John W. Duckett MD Laboratory in Pediatric Urology, Division of Pediatric Urology, Department of Urology, University of Texas Southwestern Medical Center, Dallas, TX, United States

**Keywords:** insulin-like peptide 3, relaxin-like factor, cryptorchidism, fetal, human, testis

## Abstract

**Context:** Insulin-like peptide 3 (INSL3), a protein hormone produced by Leydig cells, may play a crucial role in testicular descent as male INSL3 knockout mice have bilateral cryptorchidism. Previous studies have measured human fetal INSL3 levels in amniotic fluid only.

**Objective:** To measure INSL3 serum levels and mRNA in fetal umbilical cord blood and fetal testes, respectively.

**Design:** INSL3 concentrations were assayed on 50 μl of serum from male human fetal umbilical cord blood by a non-commercial highly sensitive and specific radioimmunoassay. For secondary confirmation, quantitative real-time PCR was used to measure INSL3 relative mRNA expression in 7 age-matched human fetal testes.

**Setting:** UT Southwestern Medical Center, Dallas, TX and Medical University of South Carolina, Charleston, SC.

**Patients or other Participants:** Twelve human male umbilical cord blood samples and 7 human male testes were obtained from fetuses 14–21 weeks gestation. Male sex was verified by leukocyte genomic DNA SRY PCR.

**Interventions:** None.

**Main Outcome Measures:** Human male fetal INSL3 cord blood serum concentrations and testicular relative mRNA expression.

**Results:** INSL3 serum concentrations during human male gestational weeks 15–20 were 2–4 times higher than published prepubertal male levels and were 5–100 times higher than previous reports of INSL3 concentrations obtained from amniotic fluid. Testicular fetal INSL3 mRNA relative expression was low from weeks 14–16, rose significantly weeks 17 and 18, and returned to low levels at week 21.

**Conclusions:** These findings further support the role of INSL3 in human testicular descent and could prove relevant in uncovering the pathophysiology of cryptorchidism.

## Introduction

An undescended testis or cryptorchidism, is one of the most common congenital anomalies, occurring in 1–4% of full-term and 1–45% of preterm male neonates (1). The pathogenesis of isolated cryptorchidism remains largely unknown but is most likely multifactorial, involving both genetic, and environmental risk factors (2). The majority of what is known of the physiology of normal testicular descent in humans is inferred from animal models, primarily rodents (2). In the male fetus, the undifferentiated gonad forms high in the abdomen and descends through the abdomen and inguinal canal to eventually reside in the scrotum by birth. Testicular descent occurs in two phases—transabdominal and transinguinal (3).

The gubernaculum, a cordlike organ spanning from the testis and vas to the scrotum, plays a key role in testicular descent. Swelling of the gubernaculum is critically important to allow enlargement of the inguinal canal and testicular passage (2) and occurs in human male embryonic development during weeks 12–17 of gestation. Rodent studies provide strong evidence that the hormone INSL3 and androgen signaling pathways are the primary contributors to gubernacular development and testicular descent (4). During embryogenesis, testicular Leydig cells are the sole producers of INSL3, which circulates and binds to relaxin family peptide receptor 2 (RXFP2) receptors on gubernacular cells, causing proliferation of the gubernaculum through alterations in extracellular matrix, Wnt, bone morphogenetic protein, β-catenin, Notch, neural and cytoskeletal/muscular patterning gene pathways (5–11). With retraction of the gubernaculum, the testis then migrates from its retroperitoneal/abdominal location to the scrotum. Mice with targeted homozygous deletions of INSL3 or RXFP2 manifest bilateral cryptorchidism, with the testes remaining high in the abdominal position, as the result of gubernacular deficiencies (4, 12, 13).

While the importance of INSL3 in murine fetal testicular descent has been demonstrated, the role of INSL3 in human fetal testicular descent and maldescent is less clearly delineated. It is true that both INSL3 and RXFP2 are highly conserved at the gene and protein levels between mice and humans. Other data from human fetal testes are limited by small numbers of samples with variance between samples. Human testis INSL3 mRNA transcripts have been detected as early as 12 weeks gestation (14) and INSL3 protein was first noted by INSL3 immunohistochemistry in a few polygonal interstitial cells at gestational week 10 (15). During the time interval of human fetal gubernacular swelling, human fetal serum testosterone concentrations decline, being significantly higher at gestational weeks 11–13 than at weeks 17–19 (14). However, to our knowledge, human fetal serum INSL3 levels have not been directly measured in fetal blood during these gestational ages. As an indirect attempt, human INSL3 levels have previously been measured in the amniotic fluid of male fetuses between gestational weeks 12–22 in three publications (16–18). In 2008, Bay et al. measured amniotic fluid INSL3 concentrations between <0.02 and 0.36 ng/ml with a non-commercial semi-competitive time resolved fluorescent immunoassay (TRFIA). In 2018, using a modified TRFIA, Anand-Ivell et al. measured human amniotic fluid INSL3 concentrations in control, hypospadic and cryptorchid males, detecting levels between undetectable and 0.9 ng/ml (18). In contrast to these fetal amniotic fluid levels, normal male puberty yields a significant progressive rise in INSL3 serum levels (19–21), ultimately achieving adult male circulating INSL3 concentrations between 0.5 and 2.0 ng/ml (22). However, since amniotic fluid is a diluted source of INSL3, we hypothesize that INSL3 concentrations in fetal cord blood will be higher than measured in amniotic fluid. These findings will thus provide a more accurate assessment of circulating INSL3 levels throughout human male fetal life and improve the understanding of the physiology of testicular descent and pathophysiology of cryptorchidism.

## Materials and Methods

### Human Samples

This study on de-identified human samples obtained from outside sources was deemed exempt by our institutional IRB. Serum umbilical cord blood samples and fetal gonads from reported normal, electively, and legally terminated pregnancies between 14 to 21 weeks gestation were provided by Advanced Bioscience Resources, Inc., (Alameda, CA). The fetal gonad tissue and umbilical cord blood were obtained from separate cases. One human adult testis sample was obtained from our institutional biobank. Clotted fetal cord blood samples (0.2–0.8 ml volume) were received in dry ice. After spinning the tubes at 2,500 rpm/min for 10 min in 4°C, the sera were drawn out and stored at −80°C for radioimmunoassay (RIA). The clotted blood in the bottom of the serum-separated gel was removed for DNA extraction. DNA was extracted from clotted whole blood samples using salting-out method (23).

### Verification of Sex in Fetal Cord Blood by SRY PCR

Male sex was verified on each fetal cord blood sample (15–20 weeks gestational age) by determination of the SRY gene by polymerase chain reaction (PCR) of leukocyte genomic DNA. Two primer pairs were used for PCR. The forward primer X1 (aatcatcaaatggagatttg) and reverse primer X2 (gttcagctctgtgagtgaaa) were used to amplify a fragment of 131 base pairs in the X chromosome. The forward primer Y11 (atgatagaaacggaaatatg) and reverse primer Y22 (agtagaatgcaaagggctc) were used to amplify a fragment of 172 base pairs in the Y chromosome (24). The PCR program included denaturing at 95°C for 3 min, followed by 30 cycles at 94°C for 40 s, 54°C for 1 min and 72°C for 50 s. PCR products were separated on a 2% agarose gel.

### INSL3 RIA

INSL3 serum concentrations were measured on 50 μl of 12 proven male serum samples (assayed once, in duplicate, or in triplicate, depending on available serum volume) by a non-commercial highly-sensitive and specific human INSL3 RIA as previously described (25). For each assay, new ^125^I tracer was made just prior to the measurements to achieve the highest sensitivity. Standard curves were obtained using human INSL3 labeled with ^125^Iodinated INSL3-mono-oxide tracer and 100 μL of 1:10,000-diluted anti-human INSL3 antibodies raised in albino rabbits. Lower and upper limits of detectability were 0.3 and 3 ng/ml. Individual samples were blinded to the person performing the assay and γ counts of the washed pellets were compared to dose-response curves of human serum INSL3.

### Verification of Fetal Sex by Gonadal Histology

Male sex was confirmed histologically on each fetal gonad (15–20 weeks gestational age) by analyzing a portion of the testis. After formalin fixation, testes were paraffin embedded, sectioned to 5 μm, stained with hematoxylin and eosin and confirmed to be testis by light microscopy.

### INSL3 mRNA qPCR

To secondarily confirm the trends of INSL3 concentration observed, INSL3 mRNA expression was assayed on 7 proven fetal testes by qRT-PCR. Total RNA was isolated using TRIzol reagent (Invitrogen, Carlsbad, CA), treated with DNase, and purified using Aurum Total RNA Mini Kit (Bio-Rad, Hercule, CA), according to manufacturers' instructions. Due to small tissue size and cDNA output, cDNA was amplified using the TaqMan PreAmp Master Mix Kit (Applied Biosystems, Foster City, CA). RNA was reverse transcribed to cDNA using SuperScript III Reverse Transcription kit (Invitrogen). Probes for INSL3 (Hs01895076_s1) or CDKN1B (Hs00153277_m1) (Life Technologies) were mixed with Taqman Universal PCR Master Mix (Applied Biosystems) and amplified in triplicate on an iCycler thermocycler (Bio-Rad). Expression levels of INSL3 were normalized to CDKN1B per the Applied Biosystems T-PreAmp uniformity reference gene assay (26) and fold differences were calculated using the ΔΔC_T_ method.

### Statistics

Because of the small number of samples, statistical analysis was limited. Shapiro-Wilk test for normality did not reject the hypothesis of normally distributed data (*p* = 0.9). Data on the serum INSL3 levels and INSL3 expression levels were presented as group mean ± standard deviation (SD) and were analyzed between groups using repeated measures ANOVA. A hierarchical linear regression model was used to test for trend of serum INSL3 levels over gestational ages 15–20 weeks. In this model, fixed effect was the measured INSL3 level and random effect was fetus, as some feti had repeated serum INSL3 measures obtained. *P* < 0.05 was considered statistically significant.

## Results

**Figure 1:**
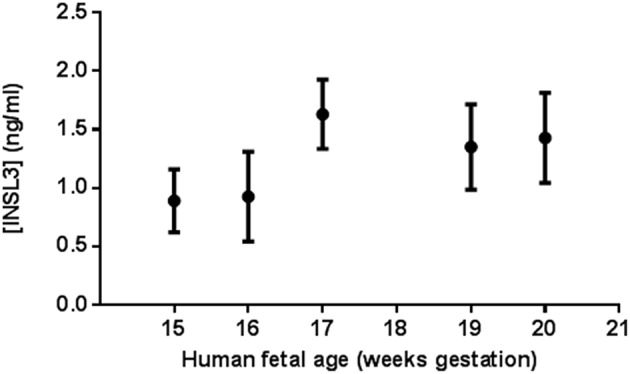
Human male fetal umbilical cord serum INSL3 concentrations during gestational weeks 15–20. Each data point represents the means ± SEM for all samples tested at that age. At gestational weeks 15, 16, 17, 19, and 20, there were 1, 3, 1, 3, and 4 fetal serum samples, respectively. No fetal cord blood was collected for age 18 weeks. All feti were assayed in duplicate or triplicate when possible. Fetus #9, 10, and 11 did not undergo repeat measure due to insufficient serum volume for repeat testing.

**Figure 2:**
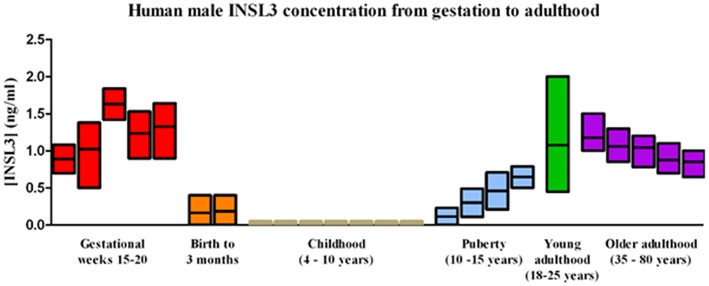
Normal human male serum INSL3 concentrations from gestation to adulthood. INSL3 concentrations were measured in fetal cord blood from gestational weeks 15–20 (this study), cord blood from newborn male infants (27–30) and in serum from 3-month-old male infants (31), prepubertal and pubertal boys (19–21), young adult males (22), and older adult males (32).

Human fetal cord blood samples from 12 males were obtained between the ages 15–20 gestational weeks (gw 15, *n* = 1; gw 16, *n* = 3; gw 17, *n* = 1; gw 19, *n* = 3; gw 20, *n* = 4) (Supplementary Figure 1). All 12 samples had measurable INSL3 levels (range 0.44–2.04 ng/ml) and all levels assayed were within the limits of detection for this RIA. Most of the feti had enough cord blood for multiple measures of serum INSL3 levels. Combining all fetal samples, the mean ± SD serum INSL3 concentration was 1.26 ± 0.43 ng/mL (Figure 1). When segregated by gestational age groups, there was no overall statistical difference found between the serum INSL3 concentrations by repeated measures ANOVA. However, there was an upward trend to the fetal serum INSL3 concentrations from 15 to 20 weeks of gestation by hierarchical linear regression (*p* = 0.02; Figures 1, 2 and Supplementary Figure 1).

**Figure 3:**
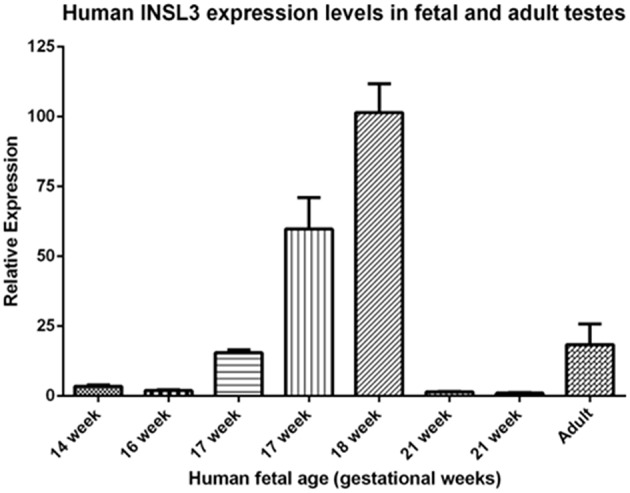
Human INSL3 expression levels in fetal and adult testes. Human INSL3 relative expression was measured by real-time quantitative PCR in fetal gonadal tissue from gestational weeks 15–21 and adult testes tissue.

A comparison of these fetal cord blood serum INSL3 concentrations with previously reported INSL3 levels reveals that fetal cord blood serum INSL3 levels are 5–100 times higher than fetal amniotic fluid levels, are 2–4 times higher than prepubertal levels, and are similar to the high levels seen in young adulthood (Figure 2 with references cited). To secondarily evaluate the trends of INSL3 concentration observed, quantitative real-time PCR was used to measure INSL3 relative expression in 7 age-matched fetal testes 14–21 weeks gestation with 1 adult testicle to serve as a comparison. INSL3 expression was detectable in all 7 fetal testes and 1 adult testes. While minimal INSL3 relative expression was observed in gestational weeks 14, 16, and 21, there was a robust peak of maximal INSL3 relative expression during weeks 17 and 18, which was over 5 times higher than observed in the adult testis (Figure 3).

## Discussion

Previous reports have shown INSL3 is only detectable in the amniotic fluid of pregnancies with a male fetus (16–18). Though these previous reports have quantified fetal INSL3 concentrations in amniotic fluid as early as week 12 of pregnancy (16–18, 31), to our knowledge this is the first report of fetal INSL3 levels obtained directly from fetal umbilical cord blood. The fetal INSL3 cord blood data herein is supported by the human fetal testis INSL3 mRNA, which we and O'Shaughnessy (14) both found to be maximal at gestational week 18. As INSL3 is produced by no other fetal organ than the testes and does not appear to be produced by the female fetus, it has the potential to be a highly specific monitor for the development and differentiation of the fetal testis (33). Our findings that INSL3 levels in fetal umbilical cord blood during human male gestational weeks 15–20 are higher than previous reports of INSL3 concentrations in amniotic fluid during gestation only strengthen the role of INSL3 in testicular descent.

During embryogenesis, Leydig cells produce INSL3, which circulates and binds to the G protein coupled receptor RXFP2 on the gubernaculum (34). This interaction results in proliferation of the embryonic gubernaculum, which in turn retracts the testis from the abdominal cavity to the scrotum. Gestational weeks 15–18, in which INSL3 levels are at their highest, coincide with gubernacular outgrowth and the initial abdominal phase of testicular descent. The findings of increased production of INSL3 after week 15 suggests that INSL3 is not only required for the transabdominal phase of descent but possibly in the inguinoscrotal phase of testicular descent, perhaps by dilating the inguinal canal (16).

It was previously believed that the second phase of testicular descent, the passage of the testes through the inguinal canal and into the scrotum, was largely under androgen control. However, a combination of androgen and INSL3 receptor antagonists in mice has shown that the remediation of testicular descent in cryptorchid mutant mice by testosterone replacement therapy required the induction by androgens of the INSL3 receptor RXFP2 (34, 35). Thus, as concluded by Ivell et al. it is reasonable to hypothesize that gonadotropin induced testicular descent may not be entirely due to the induction of testicular androgen production but rather to the stimulation of Leydig cell differentiation and thus secretion of INSL3 (34). The findings of even higher fetal INSL3 levels than previously reported via amniotic fluid at gestational weeks 18–20 further confirms the role of INSL3 in all phases of testicular descent.

In the fetal amniotic fluid level studies, the maximum concentrations of INSL3 were between 12 and 16 weeks with a decline thereafter to below detection levels (16, 17). The authors have stated that it is not clear whether this decline was secondary to a down-regulation of INSL3 expression, an increasing dilution effect, decreasing fetal skin permeability, or catabolism (33). During the first and early second trimester, amniotic fluid can be regarded to a degree as an exudate of fetal serum, however later in gestation, when the fetal skin keratinizes, the composition of amniotic fluid becomes more representative of fetal urine (36). Fetal cord blood INSL3 levels, as reported in the present study, thus provide a more accurate representation of INSL3 with levels persisting through 20 weeks gestation.

In addition, fetal cord blood INSL3 levels were found to be higher than reported INSL3 concentrations in pre-pubertal males and throughout most of adult life (Figure 2). Circulating levels of INSL3 in postnatal life are characterized by an increase early (birth to 3 months), followed by very low levels during childhood (4–10 years), with a spike in INSL3 production at puberty that leads into high levels in young adulthood before decreasing again in later adulthood (21, 22, 31, 32). As INSL3 is secreted by Leydig cells, Leydig cell differentiation influences INSL3 production and the triphasic nature of Leydig cell development coincides with the peaks of INSL3 levels seen in human life. The three populations of Leydig cells include the fetal Leydig population which reaches its maximal size around gestational weeks 14–18 (14), a perinatal population that peaks in size 3 months after birth, and the adult Leydig population which begins increasing in size at puberty.

All types of Leydig cells produce INSL3. It is believed by some that the fetal Leydig cell population responsible for fetal INSL3 production for testicular descent is completely separate from the adult Leydig population which develops from stem cells at puberty (34, 37), while others believe the human fetal Leydig cell population involutes and re-differentiates with pubertal onset. It has been suggested that INSL3 is produced constitutively, independent of acute regulation by the hypothalamo-pituitary gonadal axis, in amounts which reflect the numbers and differentiation status of the Leydig cells (33). Serum INSL3 levels may thus be a better indicator for Leydig cell function than serum testosterone concentrations in the male (38, 39). In addition, as fetal Leydig cells are a separate population from adult Leydig cells, it is possible that alterations in fetal Leydig function or INSL3 production or secretion may underlie the pathophysiology of cryptorchidism. In humans, this has been challenging to prove, with some contradictory findings. Three relatively large human studies compared serum human INSL3 cord blood levels at birth in male infants with descended and undescended testes, finding reduced INSL3 serum levels in cryptorchid boys and suggesting impaired Leydig cell function in boys with persistent cryptorchidism already in early postnatal life (27, 28, 31). The major criticism of these three studies is that serum levels at birth or 3 months postnatally may not reflect second trimester levels. To address this concern, Jensen et al studied 270 cryptorchid and 300 control second trimester amniotic fluid samples' INSL3 and phthalate levels and found no clear association (40). In addition to other estrogenic and anti-androgenic compounds, fetal testes cells exposed in culture to paracetamol and ketoconazole have decreased INSL3 levels which may be the mechanism by which analgesics, and possibly other endocrine disruptors, increase the risk of cryptorchidism (41).

Nevertheless, serum levels of a hormone do not necessarily correlate with required tissue levels for proper development and function. For example, it is known that postnatal rat testosterone concentrations assayed from either the testicular vein (42) or the testicular interstitial fluid (43) are 20 times and 100 times higher than systemic circulating serum concentrations, respectively. In addition, varying serum and tissue testosterone concentrations on fetal mammalian paratesticular tissues (directly on the vas, epididymis, seminal vesicles, gubernaculum, and indirectly via 5-alpha-reductase/dihydrotestosterone on the penis/urethra, prostate, and scrotum) via endocrine and paracrine mechanisms mediate the phenotypic spectrum of male birth defects seen in disorders of sex development (44–46). Similarly, it has been shown that rat fetal testicular INSL3 mRNA expression probably requires at least 40% reduction before a change in phenotype occurs (47). Hence, human fetal amniotic fluid and even human fetal cord blood INSL3 concentrations likely do not truly measure and reflect the threshold of INSL3 fetal tissue concentration required to induce tissue level maldevelopment of the gubernaculum and maldescent of the testis during fetal life. They are indirect measures at best.

The main limitation to this study is the small sample size secondary to limited access to umbilical cord blood from human fetuses aged 12–20 gestational weeks. A second potential limitation of this study is the INSL3 assay used. Nowadays, INSL3 concentrations can be measured by several assays, including RIA, EIA, ELISA, TRFIA, and LC-MS/MS. Our INSL3 RIA assay was performed several years ago to generate our INSL3 data, explaining the older methodology. As a result, this older assay has not actually been fully validated, hence it is not known if the assay cross-reacts with other plasma components. The plasma samples did parallel the standard curve for both fetal and adult plasma samples, suggesting that the endogenous INSL3 peptide is similar in adult and fetal blood. Unfortunately, the quantity of each serum sample was insufficient to permit replication of the serum Insl3 concentrations by one of the newer assays that have a lower limit of detection than our RIA. Given the lower and upper limits of detectability of our RIA were 0.3 and 3 ng/ml, the RIA is at least one order of magnitude less sensitive than the assays in current use, which have lower limits of detection ranging from 0.01 to 0.06 ng/ml (18, 19, 27–29, 48, 49). Less sensitivity can increase intra-assay coefficient of variation. While this would be most problematic if the serum levels were quite low, the mean serum umbilical cord INSL3 levels were well within the midrange of the limits of detection of the RIA assay and the mRNA data is corroborative. Additionally, while the fetuses were labeled as normal based on prenatal investigation prior to elective termination, the ultimate phenotype as a term infant is unknown, and the study could theoretically have included samples that might have gone on to develop cryptorchidism or a disorder of sex development, thus skewing results. Lastly, we did not measure fetal cord serum levels of H2 relaxin (RNL2). While there is theoretical concern that H2 relaxin is able to activate the RXFP2 receptor given this occurs *in vitro*, it occurs only at supra-physiological concentrations which are unlikely to occur in the fetus since no major relaxin expression has been observed on tissue microarray studies before puberty. Thus, most evidence suggests H2 relaxin is unlikely to play a major role in testicular descent (50).

## Conclusions

Human fetal levels of INSL3 have previously only been studied in amniotic fluid, which underestimated the circulating concentration of INSL3. This study of INSL3 levels obtained from fetal cord blood and testes indicates that INSL3 serum concentration during male fetal life is 5–100 times higher than INSL3 levels observed in amniotic fluid, is 2–4 times higher than serum levels of prepubertal boys, and is amazingly similar to that of young adult men. These findings lend further support to the role of INSL3 in human testicular descent during fetal life in males.

## Author Contributions

All authors listed have made a substantial, direct and intellectual contribution to the work, and approved it for publication.

### Conflict of Interest Statement

The authors declare that the research was conducted in the absence of any commercial or financial relationships that could be construed as a potential conflict of interest.

## Supplementary Material

Supplementary Figure 1 Human Fetal umbilical cord serum INSL3 concentrations: raw data. Human umbilical cord serum INSL3 concentrations raw data, reporting individual and repeated measures from each fetus by gestational age.

## Abbreviations

DNA: Deoxyribonucleic acid
gw: Gestational week
INSL3: Insulin-Like Peptide 3
PCR: Polymerase Chain Reaction
qRT-PCR: Quantitative reverse transcription-polymerase chain reaction
RIA: Radioimmunoassay
RXFP2: Relaxin family peptide receptor 2
mRNA: Messenger Ribonucleic acid
EIA: Enzyme immune assay
ELISA: Enzyme Linked Immunosorbent Assay
SRY: Sex-determining region of the Y gene
TRFIA: Time-Resolved Fluorescence Immunoassay
SD: Standard deviation
LC-MS/MS: Liquid Chromatography with tandem Mass Spectrometry.
