# Supplementary material for: Insulin-Like Peptide 3 (INSL3) Serum Concentration During Human Male Fetal Life
Source: Front Endocrinol (Lausanne). 2019 Sep 4;10:596. doi: 10.3389/fendo.2019.00596 (PMC6737488; doi:10.3389/fendo.2019.00596)
Supplement: Supplementary Figure 1 — Human Fetal umbilical cord serum INSL3 concentrations: raw data. Human umbilical cord serum INSL3 concentrations raw data, reporting individual and repeated measures from each fetus by gestational age. [file Data_Sheet_1.PDF]

Human Fetal Umbilical Cord Serum INSL3 Concentrations: Raw Data

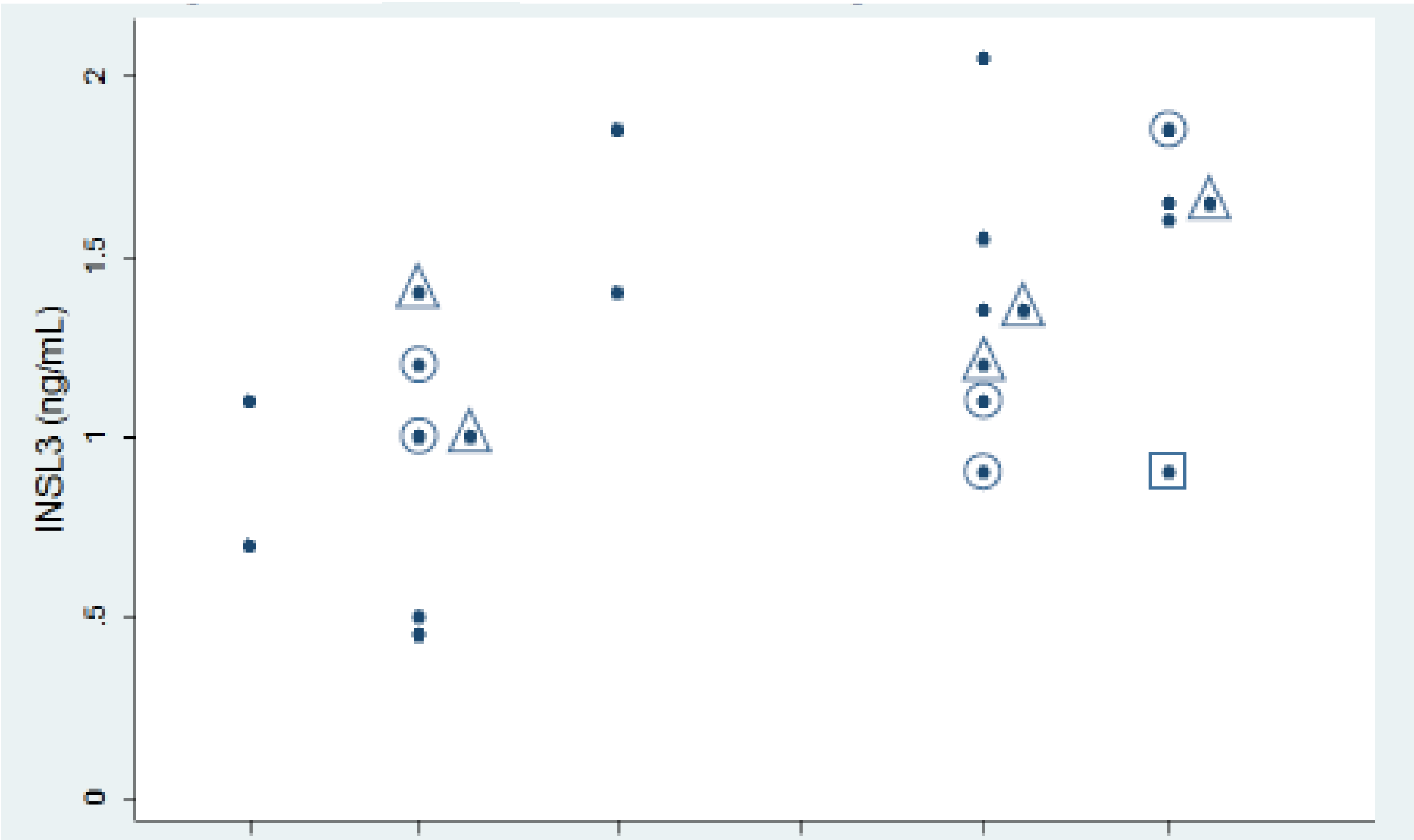

Age (gest wks):	15	16	17	18	19	20
# Feti:	1	3	1	0	3	4

Gestational age (weeks)	Number of feti tested	Fetus #	INSL3 [ng/ml]
15	1	1	0.70
		1	1.08
16	3	2	0.44
		2	0.50
		3	1.02
		3	1.38
		4	1.00
		4	1.20
17	1	5	1.42
		5	1.84
18	0		
19	3	6	1.53
		6	1.33
		6	2.04
		7	1.19
		7	1.37
		8	0.90
		8	1.10
20	4	9	1.66
		10	1.84
		11	0.90
		12	1.6
		12	1.64

*All feti were assayed in duplicate or triplicate except Fetus # 9, 10 and 11, which did not undergo repeat measure
